# The effect of calorie restriction on mouse skeletal muscle is sex, strain and time-dependent

**DOI:** 10.1038/s41598-017-04896-y

**Published:** 2017-07-11

**Authors:** Luisa Boldrin, Jacob A. Ross, Charlotte Whitmore, Bruno Doreste, Charlotte Beaver, Ayad Eddaoudi, Daniel J. Pearce, Jennifer E. Morgan

**Affiliations:** 1 Dubowitz Neuromuscular Centre, Molecular Neurosciences Section, Developmental Neurosciences Programme, UCL Great Ormond Street Institute of Child Health, 30 Guilford Street, London, WC1N1EH UK; 2 UCL Institute of Healthy Ageing, University College London, London, WC1E 6BT UK; 3 Flow Cytometry Core Facility, UCL Great Ormond Street Institute of Child Health, Camelia Botnar Laboratories, Great Ormond Street Hospital, 85 Lamb’s Conduit, London, WC1N 3JH UK

## Abstract

Loss of skeletal muscle mass and function occurs with increasing age. Calorie restriction (CR) increases the lifespan of C57Bl/6 mice, but not in the shorter-lived DBA/2 strain. There is some evidence that calorie restriction reduces or delays many of the age-related defects that occur in rodent skeletal muscle. We therefore investigated the effect of short (2.5 month) and longer term (8.5 and 18.5 months) CR on skeletal muscle in male and female C57Bl/6 and DBA/2 mice. We found that short-term CR increased the satellite cell number and collagen VI content of muscle, but resulted in a delayed regenerative response to injury.Consistent with this, the *in vitro* proliferation of satellite cells derived from these muscles was reduced by CR. The percentage of stromal cells, macrophages, hematopoietic stem cells and fibroadipogenic cells in the mononucleated cell population derived from skeletal muscle was reduced by CR at various stages. But overall, these changes are neither consistent over time, nor between strain and sex. The fact that changes induced by CR do not persist with time and the dissimilarities between the two mouse strains, combined with sex differences, urge caution in applying CR to improve skeletal muscle function across the lifespan in humans.

## Introduction

Skeletal muscle consists of postmitotic multinucleated myofibres that are specialized contractile cells. Myofibres form during development by fusion of muscle precursor cells (myoblasts) and continue to grow after birth. Muscle growth, repair and regeneration are mediated by satellite cells, muscle-specific stem cells that are located under the basal lamina of myofibres, from which myoblasts are derived.

Muscle mass and function are not maintained beyond middle age. Sarcopenia is associated with a lack of muscle strength, leading to reduced posture and mobility, increased risk of falls and reduced quality of life in old age^1^. Myofibres from aged muscle have increased susceptibility to contraction-induced injury and a reduction in force generation. Other age-related changes in skeletal muscle include mitochondrial abnormalities^2^, changes in protein synthesis and degradation^3^, increased inflammation^4^, apoptosis and elevated levels of oxidative damage^5^.

Satellite cell numbers are reduced with increasing age^6–8^; in addition, other muscle-resident cells, including inflammatory cells, macrophages, pericytes, endothelial cells, myoendothelial cells, fibroblasts, capillaries and motor nerve terminals may be affected by ageing. The ability of skeletal muscle to regenerate is diminished in old age, but this may be a consequence of an impairment of the environment, rather than the stem cells themselves. Aged muscles regenerate well when either grafted into a young host, or exposed to a young systemic environment^9–11^ and satellite cells from aged mice can regenerate and self-renew as well as those derived from a young donor, when grafted into a permissive young host environment^7, 12^.

Rejuvenating satellite cell function in ageing muscle could produce more cells capable of maintaining and repairing damaged myofibres and for generating new fibres to replace those lost with age.

Calorie restriction (CR), defined as a diet low in calories without under-nutrition, extends lifespan in rodents and appears to do the same in humans^13^. It also reduces the incidence of age-related diseases in humans (reviewed^14^) and in mice (reviewed^15^). However, the mechanism by which CR extends lifespan is not completely understood: it may activate stress responses that increase the chances of surviving adversity^16^, or reduce the metabolic rate, leading to a decline in oxidative damage. CR also leads to hormonal changes and to a reduction in body temperature that in turn affects ageing^17^. A recent paper^18^ has however challenged the notion of a direct correlation between lifespan extension, health, and CR, regardless of the context (strain, sex and extent of CR). They used male and female C57BL/6 J and DBA2/J mice, given 20% or 40% CR and concluded that for CR to have a beneficial effect, it must cause maintenance of healthy and functional mitochondria and active autophagy. Such changes led to improvements in carbohydrate and lipid metabolism, allowing metabolic flexibility and preservation of body fat into old age.

CR in rodents appears to either reduce, or delay the onset of many age-related changes in skeletal muscle^19^. Cerletti *et al*. showed that short-term (3-month) CR increased satellite cell numbers and activity in both young and old male C57Bl/6 mice^20^. Satellite cells displayed increased mitochondrial abundance and expression of factors involved in regulating self-renewal as Sirtuin1, FOXO3a and activated Notch^21–23^. Furthermore, CR improved the regenerative potential of satellite cells *in vivo* ^20^.

However, it appears that there is a differential response to CR between different strains of mice. In C57Bl/6 mice, the lifespan is extended by CR, whereas in the shorter-lived DBA/2 strain, it does not seem to be the case^24^. There is also evidence that satellite cells from DBA/2 mice contribute less efficiently to muscle regeneration than those from C57Bl/10 mice, but no direct comparison with C57Bl/6 mice was made^25^. It is not clear whether these strain-specific responses translate to alterations in muscle stem cell ageing, particularly within the context of CR.

We therefore investigated the effect of short (3 month) and longer term (9 and 19 months) CR on skeletal muscle in male and female C57Bl/6 and DBA/2 mice. We found that CR led to changes in the satellite cell number within skeletal muscle and in the proliferation capacity of satellite cells extracted from muscles and cultured *in vitro*. In addition, the proportions of cell populations within skeletal muscle, the regenerative response of skeletal muscle to injury and the extent of fibrosis within muscles were modulated in CR mice. However, these changes were strain, sex and age dependent.

## Materials and Methods

Experimental procedures were carried out in the Biological Services Unit, University College London, in accordance with the Animals (Scientific Procedures) Act 1986. Experiments were approved by the UCL Animal Welfare and Ethical Review Body. Experiments were performed under Home Office licence number 70/7086.

Male and female C57Bl/6 and DBA/2 mice were purchased from Charles River and either subjected to calorie restriction^26^, or fed *ad libitum* (controls). Mice were housed individually. CR was initiated at 14 weeks of age at 10% restriction, increased to 25% restriction at 15 weeks and to 40% restriction at 16 weeks where it was maintained throughout the life of the animal. Mice were weighed at monthly intervals and their weights recorded.

**Figure 1:**
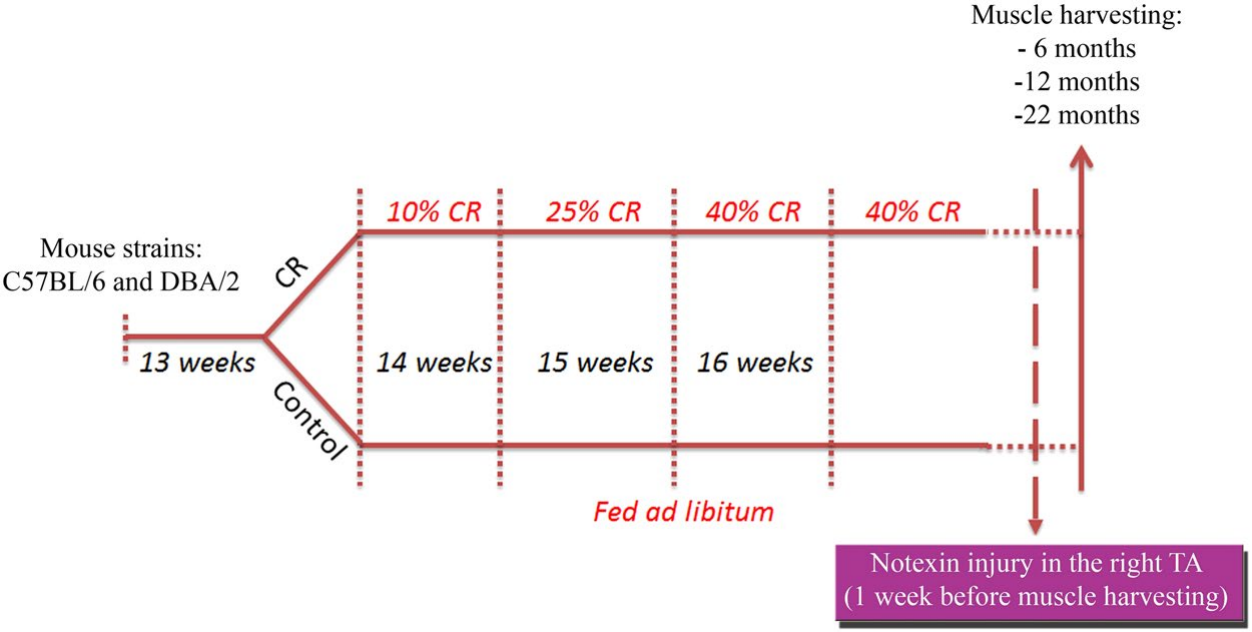
Design of the experimental plan. C57Bl/6 and DBA/2 mice were all individually housed at 13 weeks and calorie restriction was started in the calorie restricted (CR) group at 14 weeks of age. 10% calorie restriction was applied the first week, 25% calorie restriction the week after (mice aged 15 weeks) and 40% calorie restriction was started from 16 weeks of age until the end of the experiments. Control mice were fed ad libitum for all the length of the experiment. In the injury cohort, mice had their right *tibialis anterior* (TA) injected with notexin and experiments were terminated one week later, when mice reached the age of 6, 12 or 22 months (after 2.5, 8.5 or 18.5 months CR).

We analysed mice at three timepoints: 6, 12 and 22 months of age, corresponding to 2.5, 8.5 and 18.5 months of CR, respectively (Fig. 1).

### Response of muscles of calorie-restricted and age-matched control mice to injury

The *tibialis anterior* (TA) muscles of the right legs (RTA) of male and female CR and male and female control mice at each timepoint were injured by injection of *10 µl Notechis scutatus* notexin (Latoxan) (10 µg/ml), a myotoxin that destroys muscle fibres, whilst sparing blood vessels and other cell types, including satellite cells^27^. Contralateral, left leg muscles (LTA) were used as non-treated controls. Muscles were removed at one week after injury^27^. Number of Pax7+ cells, fibrotic area and myofibre cross-sectional area (CSA) were measured as described below.

### Skeletal muscle analyses

The TA muscles were removed from left hindlimbs of mice at each time point for histological analysis. 7 µm transverse cryosections were cut from these muscles.

Muscle sections were stained with a mouse monoclonal antibody to Pax7 (Developmental Studies Hybridoma Bank) that identifies satellite cells and co-stained with a rabbit polyclonal antibody to laminin to identify the basal lamina (Sigma)^28^. Other sections were stained with a rabbit polyclonal antibody to collagen VI (Abcam) to identify fibrotic areas^29^. The number of Pax7 +ve cells under the basal lamina were counted and recorded in 6 random images, and presented as number per 100 fibres.

Fibrosis was quantified as the percentage of collagen VI positive area on representative transverse sections of each muscle^29^. Collagen VI is a common marker of fibrosis (as used previously by^29–35^) and correlates well with other methods such as Sirius Red staining^36^.

Myofibre CSA was also measured on laminin-stained sections. Digital analyses were performed by using Image J software.

### Flow cytometry

TA and gastrocnemius muscles from both legs of male and female calorie restricted and control mice at each timepoint were disaggregated and sorted by flow cytometry to obtain populations enriched for stem cells present in skeletal muscle, using the following markers.

CD45 + (haematopoietic stem cells, HSCs), CD68 + (macrophages), Lin-/CD31 + /Sca1 + (endothelial cells), Lin-/CD31-/Sca1 + (stromal cells), Lin-/CD31-/Sca1 + /CD34 + /integrin-α7- (fibro/adipogenic precursors, FAPs)^37, 38^.

Supplementary Figure 1 shows the FACS plots of cells derived from a female control C57 mouse at 22 months, to describe how the FACS plots were analysed. Data are presented as the percentage of each cell type within the total live/singlet cell population.

### Clonal cultures

The ability of satellite cells to proliferate and to differentiate was assessed by preparing isolated myofibres from EDL muscles from mice at the 12 and 22 month timepoints, stripping satellite cells from the myofibres and plating them at 0.5 cells/well of a 96 well plate on Matrigel (0.1 mg/ml) in proliferation medium (DMEM/10% FBS/0.5% chick embryo extract). The number of cells per clone was analysed on day 7 after plating.

### Statistical analyses

A one-way ANOVA was used to analyse >2 groups at a time, followed by Bonferroni test to confirm individual statistical differences. T-test was used to compare two groups presenting normal distribution and similar variances. Groups were considered significantily different when p < 0.05 (*), <0.01 (**) and <0.001 (***). GraphPad software was used for the ANOVA analyses.

A simple multiple regression analysis was also performed using IBM’s SPSS (version 24) statistical analysis package. In each analysis the assumptions required to implement a linear regression were checked. A summary of the results is shown in Supplementary Tables 2 and 3.

## Results

### Calorie restriction increases satellite cell number in non-injured muscles and in muscles that have regenerated in response to injury

To determine whether CR has an effect on satellite cell number or function, we quantified satellite cells in non-injured muscles and in regenerated muscles one week after notexin-induced injury.

### C57Bl/6 mice

#### Non-injured control muscles

At 6 months, of age, although there was no difference in satellite cell number between either male CR and control and female CR and control, when data from both sexes were pooled, there were significantly more satellite cells in the CR than in the control non-injured muscles (p < 0.01). At 12 months, CR males had significantly fewer satellite cells compared to controls (p < 0.05).

#### Injured muscles

**Figure 2:**
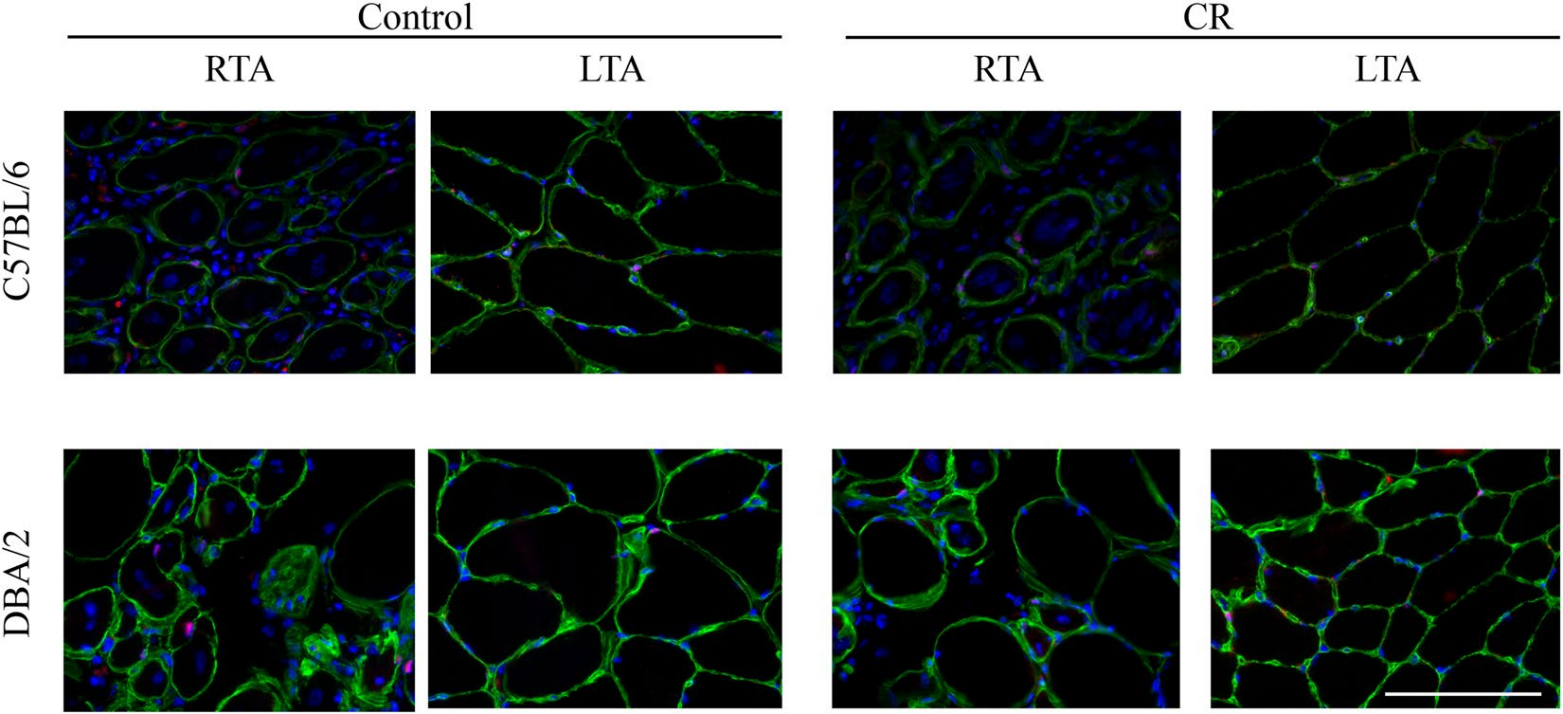
Satellite cell number in injured and non-injured control and CR muscles. Representative images of *tibialis anterior* (TA) muscles immunostained with antibodies against laminin (detecting the basal lamina, in green) and against Pax7 (detecting satellite cells, in red, beneath the basal lamina). Nuclei were counterstained with DAPI. Images are taken from both C57Bl/6 and DBA/2 22 month old mice, and both calorie restricted (CR) and control groups. Right TA (RTA) muscles were injected with notexin one week before the end of the experiment and the left TA (LTA) muscles were uninjured. Scale bar: 50 µm.

At 6 months of age, there was a significant increase in satellite cell numbers one week after injury in CR (p < 0.001) mice, compared to their non-injured counterparts. There were significantly more satellite cells in CR compared to control mice (P < 0.01 when sexes pooled; P < 0.05 for males, not significant for females) at this time point. At 12 months, there were significantly fewer satellite cells in CR males than control males (P < 0.05) (Fig. 2 and Table 1).

**Table 1:** Satellite Cells.

	**Control injured (RTA)**			**Control uninjured (LTA)**			**CR injured (RTA)**			**CR uninjured (LTA)**		
	**Mean**	**± SEM**	**n**	**Mean**	**± SEM**	**n**	**Mean**	**± SEM**	**n**	**Mean**	**± SEM**	**n**
**6 months**												
*Pooled sexes*												
C57BL/6	2.91	0.34	12	1.38 *	0.14 *	12 *	6.42^&^	1.12^&^	12^&^	2.66 ^&^	0.41 ^&^	12 ^&^
DBA/2	5.83^	0.67^	12^	2.66*^	0.35*^	12*^	7.92^~^	1.13^~^	8^~^	2.86^~^	0.56^~^	8^~^
*Males*												
C57BL/6	3.20	0.56	6	1.39	0.11	6	8.27^#^	1.58^#^	6^#^	2.61^#^	0.64^#^	6^#^
DBA/2	5.41	0.94	6	2.50	0.60	6	7.41	1.87	4	2.61	1.13	4
*Females*												
C57BL/6	2.62	0.40	6	1.37^°°^	0.27^°°^	6^°°^	4.57	1.30	6	2.70	0.57	6
DBA/2	6.24	1.01	6	2.81^°°^	0.39^°°^	6^°°^	8.42	1.52	4	3.11	0.39	4
**12 months**												
*Pooled sexes*												
C57BL/6	4.88	0.60	8	3.05	0.39	11	3.30	0.48	8	2.41	0.36	12
DBA/2	4.47	0.85	10	1.93	0.28	13	2.55	0.52	5	1.62	0.36	10
*Males*												
C57BL/6	5.05^+^	0.57^+^	5^+^	3.71^♢^	0.48^♢^	6^♢^	3.32^+^	0.50^+^	5^+^	2.07^♢^	0.42^♢^	6^♢^
DBA/2	6.32	1.56	4	1.43	0.25	6	2.30	1.02	2	1.90	0.71	5
*Females*												
C57BL/6	4.60	1.50	3	2.26	0.44	5	3.25	1.14	3	2.75	0.59	6
DBA/2	3.24	0.65	6	2.36	0.42	7	2.73	0.73	3	1.35	0.24	5
**22 months**												
*Pooled sexes*												
C57BL/6	4.10	0.64	9	1.92	0.28	10	4.80	1.11	7	2.06	0.25	10
DBA/2	5.20	1.18	5	2.37	0.51	6	2.98	0.75	5	1.39	0.20	6
*Males*												
C57BL/6	5.24	0.82	5	2.46	0.27	6	5.83	2.60	3	2.15	0.14	4
DBA/2	4.69	0.49	3	2.71	0.64	4	3.94	0.84	3	1.64	0.26	3
*Females*												
C57BL/6	2.68	0.36	4	1.11	0.22	4	4.04	0.68	4	2.00	0.42	6
DBA/2	5.96	3.49	2	1.70	0.83	2	1.54	0.19	2	1.14	0.28	3

Quantification of satellite cells in injured and un-injured control and CR muscles. Satellite cells were counted as Pax7 + ve cells below the basal lamina of tibialis anterior (TA) myofibres on transverse cryosections of calorie restricted (CR) or control mice. The right tibialis anterior (RTA) was injured by notexin injection one week before muscle harvesting; the left TA muscle (LTA) was uninjured. Number of satellite cells is presented as number per 100 fibres. Data are presented as mean ± standard error of the mean (SEM), and n numbers are specified in each case. Superscript symbols and underlinings are used to match measurements that are significant between each other.

### DBA2 mice

#### Non-injured control muscles

There was no significant difference in the number of satellite cells/100 myofibres in non-injured muscles of CR male or female 6 month old mice.

#### Injured muscles

There was a significant increase in satellite cell numbers one week after injury in control (p < 0.01) and CR (p < 0.001) 6 month old mice, compared to their non-injured counterparts (Fig. 2 and Table 1), although no effects of diet were noted.

The data suggest that CR is able to modulate the availability of satellite cells (increased at 6 months and decreased at 12 months, in both injured and non-injured muscles); however these changes are sex and strain dependent, and are no longer discernible by 22 months.

### Comparison between C57Bl/6 and DBA/2 mice

At 6 months of age, DBA/2 mice had more satellite cells than C57Bl/6 mice in control non-injured muscles (p < 0.01 in pooled data and p < 0.05 in females, see Table 1). At the later time points of 12 and 22 months, there were no significant strain differences with regard to satellite cell number, in either sex or diet group. This also indicates that at these timepoints, the satellite cell response to injury was less than in younger mice, as might be expected (reviewed^39^).

### Calorie restriction increases the amount of collagen VI in skeletal muscle

There is known to be an increase in skeletal muscle fibrosis in mice with increasing age (reviewed^40, 41^). To determine whether CR affects skeletal muscle fibrosis, collagen VI was quantified in CR and control muscles at different ages. Collagen VI has a critical effect on skeletal muscle function, as it is a component of the satellite cell niche^42^ and its loss is associated with Bethlem and Ullrich muscular dystrophies (reviewed^43^), so we were particularly interested in any changes in this particular constituent of the ECM in response to CR.

### C57Bl/6 mice

#### Non-injured control muscles

**Figure 3:**
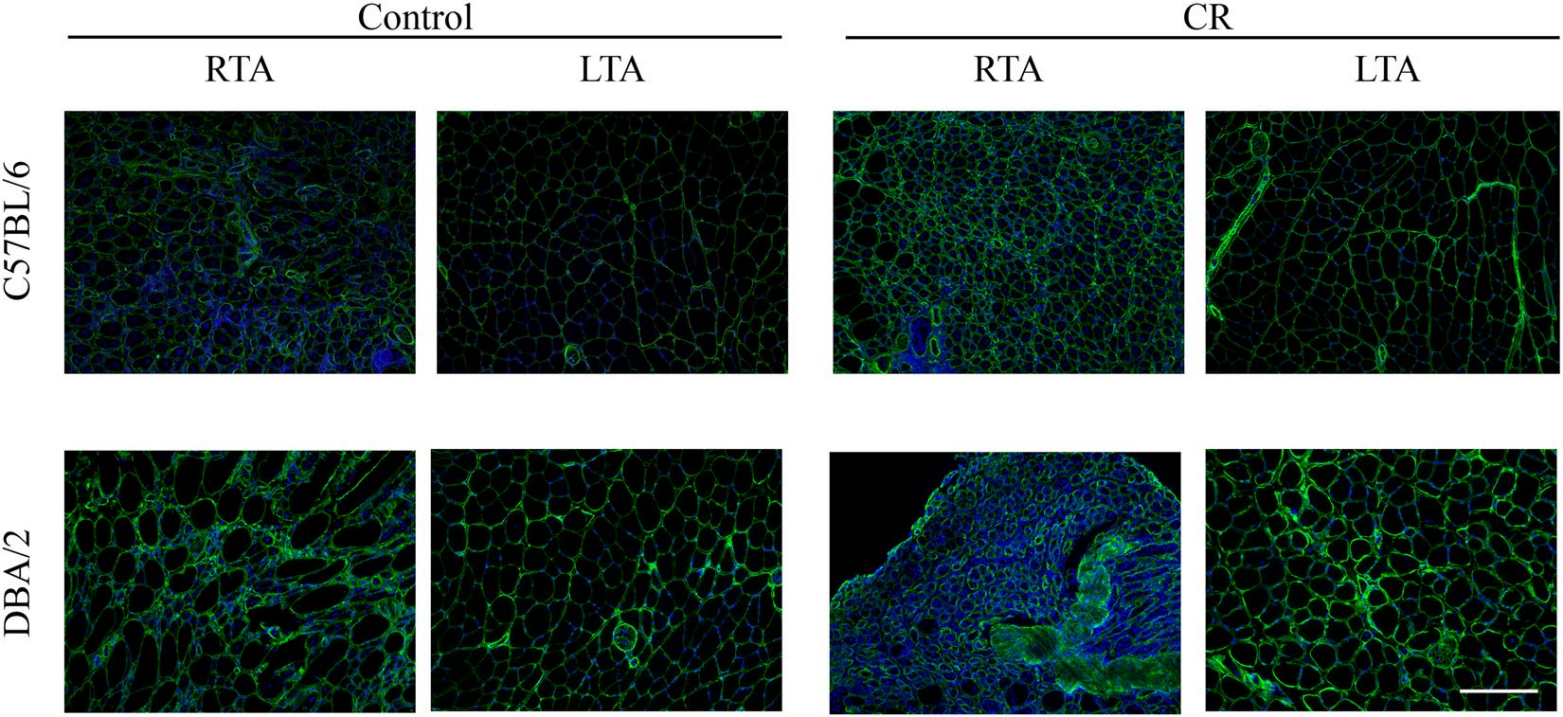
Collagen VI positive area in injured and non-injured control and CR muscles. Representative images of *tibialis anterior* (TA) muscles immunostained with antibody against collagen VI, to detect fibrotic areas. Nuclei were counterstained with DAPI. Images are taken from both C57Bl/6 and DBA/2 22 month old mice, for both calorie restricted (CR) and control groups. Right TA (RTA) muscles were injected with notexin one week before the end of the experiment while the left TA (LTA) muscles were uninjured. Bar size: 100 µm.

At 6 months of age, there was a significant increase in fibrosis in the non-injured muscles of CR mice compared to control mice (p < 0.05) (Fig. 3 and Table 2), but there was no significant difference at 12 months of age.

**Table 2:** Collagen VI (% of fibrotic area).

	Control injured (RTA)			Control uninjured (LTA)			CR injured (RTA)			CR uninjured (LTA)		
	Mean	± SEM	n	Mean	± SEM	n	Mean	± SEM	n	Mean	± SEM	n
**6 months**												
*Pooled sexes*												
C57BL/6	21.94	1.30	12	9.78 *	0.43 *	12 *	26.21°	1.17°	12°	11.28*°	0.31*°	12*°
DBA/2	26.19^&^	2.17^&^	11^&^	14.91^&^	1.29^&^	10^&^	24.11	0.88	9	17.65	1.76	9
*Males*												
C57BL/6	20.95	2.18	6	9.54	0.42	6	26.66	2.36	6	11.27	0.48	6
DBA/2	29.14	3.37	6	16.00	2.43	5	23.74	1.03	6	18.13	1.91	6
*Females*												
C57BL/6	22.93	1.52	6	10.02	0.79	6	25.76	0.59	6	11.30	0.45	6
DBA/2	22.64	1.79	5	13.82	1.01	5	24.86	1.87	3	16.67	4.26	3
**12 months**												
*Pooled sexes*												
C57BL/6	22.17^$^	2.40^$^	8^$^	13.33^$^	0.93^$^	11^$^	23.10^^^	1.74^^^	8^^^	12.57^^^	0.94^^^	12^^^
DBA/2	18.78^#^	1.37^#^	10^#^	13.95^#^	0.80^#^	13^#^	21.45^♢^	1.12^♢^	6^♢^	14.88^♢^	1.05^♢^	11^♢^
*Males*												
C57BL/6	18.81	0.86	5	12.45	0.98	6	24.50	4.11	5	14.05	1.95	6
DBA/2	14.09	0.28	4	12.05	0.43	6	20.69	1.97	3	14.05	1.52	6
*Females*												
C57BL/6	26.37	4.75	3	14.20	1.55	5	22.06	1.16	3	11.59	0.81	6
DBA/2	21.03	1.19	6	15.39	1.02	7	22.2	1.37	3	15.88	1.47	5
**22 months**												
*Pooled sexes*												
C57BL/6	15.41^>^	1.25^>^	8^>^	11.97^>^	0.45^>^	11^>^	17.34	0.90	8	12.60	0.56	12
DBA/2	15.99	1.09	5	10.56	0.34	6	16.73^ð^	2.83 ^ð^	5 ^ð^	11.81 ^ð^	0.61 ^ð^	6 ^ð^
*Males*												
C57BL/6	14.91	1.63	5	11.85	0.77	6	17.91	1.38	5	13.35	0.68	6
DBA/2	14.95	1.33	3	10.73	0.51	4	15.84	4.26	3	12.25	1.24	3
*Females*												
C57BL/6	16.24	2.27	3	12.10	0.47	5	16.39	0.70	3	11.85	0.83	6
DBA/2	17.57	1.53	2	10.23	0.08	2	18.06	4.73	2	11.37	0.37	3

Percentage of fibrotic area in injured and non-injured control and CR muscles. Collagen VI positive area (expressed as percentage of fibrotic area) was quantified in transverse TA muscles sections of calorie restricted (CR) or control mice. The right TA (RTA) was injured by notexin injection one week before muscle harvesting; the left TA (LTA) was uninjured. Data are presented as mean ± standard error of the mean (SEM), and n numbers are specified in each case. Superscript symbols and underlinings are used to match measurements that are significant between each other.

#### Injured muscles

As expected, injured muscles had more fibrosis than non-injured muscles in control (p < 0.001) and CR (p < 0.001) 6 month old mice. At 12 months of age, there was again a significant increase in fibrosis in injured than in non-injured muscles of CR and control (p < 0.001 and p < 0.05 respectively) mice. At 22 months of age, more fibrosis was found in injured muscles compared to non-injured muscles of control mice (p < 0.05).

### DBA/2 mice

#### Non-injured control muscles

At 6 and 12 months of age, there was no significant difference in fibrosis in the non-injured muscles of CR mice compared to control mice.

#### Injured muscles

As expected, injured muscles had more fibrosis than non-injured muscles in control 6 month old mice (p < 0.001). At 12 months of age, there was again a significant increase in fibrosis in injured than in non-injured muscles of CR and control (p < 0.01 and p < 0.05 respectively) mice. At 22 months of age, in CR mice, injured muscles had more fibrosis compared to non-injured muscles ((p < 0.001).

These data show that when muscles are not significantly fibrotic (young mice), the effect of CR is strain dependent.

### Calorie restriction reduces muscle fibre size 7 days after muscle injury, but this is strain, sex and age-dependent

In order to determine whether CR has an effect on muscle regeneration, we quantified myofibre size in uninjured muscles and in muscles that had regenerated for 7 days in response to notexin.

### C57Bl/6 mice

#### Non-injured muscles

There was no significant difference in muscle fibre size in the non-injured muscles of CR mice compared to control mice in both males and females at all three timepoints.

#### Injured muscles

**Figure 4:**
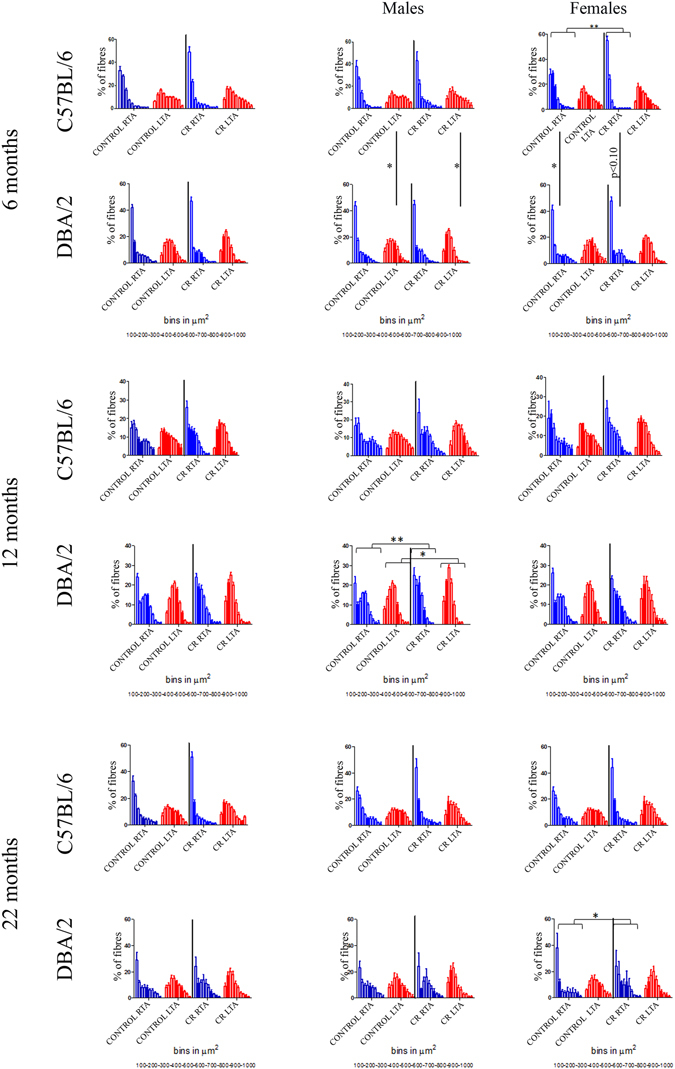
Distributions of the myofibre cross sectional area (CSA) in injured and un-injured control and CR muscles. Graphs show myofibre size distribution at 6, 12, 22 months time points, in all the mice first and then in males and females separately, of injured RTA (blue) or non-injured LTA (red) in both C57Bl/6 and DBA/2 strains. Graphs show mean +/− SEM.

There was a significant shift to muscle fibres of smaller diameter in notexin-injured muscles of 6 month old female CR mice compared to control female mice at this timepoint (p < 0.01, Fig. 4, Supplementary Table 1), indicating either a greater number of newly-regenerated fibres, or that the regenerating fibres in CR mice take longer to mature, due to the reduction in nutrients.

### DBA/2 mice

#### Non-injured control muscles

In male mice at the 12 month timepoint, fibres were of smaller diameter in non-injured muscles of CR compared to control mice (p < 0.05, Fig. 4).

#### Injured muscles

In male mice at the 12 month timepoint, fibres were of smaller diameter in injured muscles of CR compared to control mice (p < 0.01, Fig. 4). In female mice at the 22 month timepoint, there was a significant shift to smaller diameter fibres following muscle injury in CR compared to control mice, suggesting a delay in muscle regeneration (p < 0.05, Fig. 4, Supplementary Table 1).

### Comparison between C57Bl/6 and DBA/2 mice

Muscle fibre size is significantly less in non-injured muscles of DBA/2 males than in C57Bl/6 males at 6 months of age (p < 0.01 in both control and CR muscles, Supplementary Figure 2). 7 days after notexin-induced injury, muscle fibres were smaller in muscles of female C57Bl/6 than female DBA/2 mice (p < 0.05 in control muscles and p < 0.10 in CR muscles, Fig. 4, Supplementary Table 1).

A simple multiple regression analysis (Supplementary Table 2) shows that whether the muscle was injured or not was the main contributing factor to determining median fibre size (ß = 0.624; p < 0.0005) with non-injured muscles having a larger fibre size than injured muscles. Fibre size increases with age (ß = 0.226; p < 0.0005) and is reduced by increasing time on CR (ß = −0.248; p < 0.0005). However, median CSA is also dependent on strain (ß = −0.132; p < 0.01) with DBA mice having a smaller fibre size than C57Bl/6 mice. Finally, females were shown to have a smaller median CSA than males (ß = −0.109; p < 0.05). This confirms that calorie restriction reduces fibre size, although strain, sex, and age also have an influence on fibre size.

Overall these data suggest that CR may induce muscle atrophy and delay muscle regeneration/fibre size expansion, but this seems to be strain, sex and age dependent.

### Satellite cells from calorie restricted mice exhibit reduced proliferation *in vitro*

#### C57Bl/6 mice

**Figure 5:**
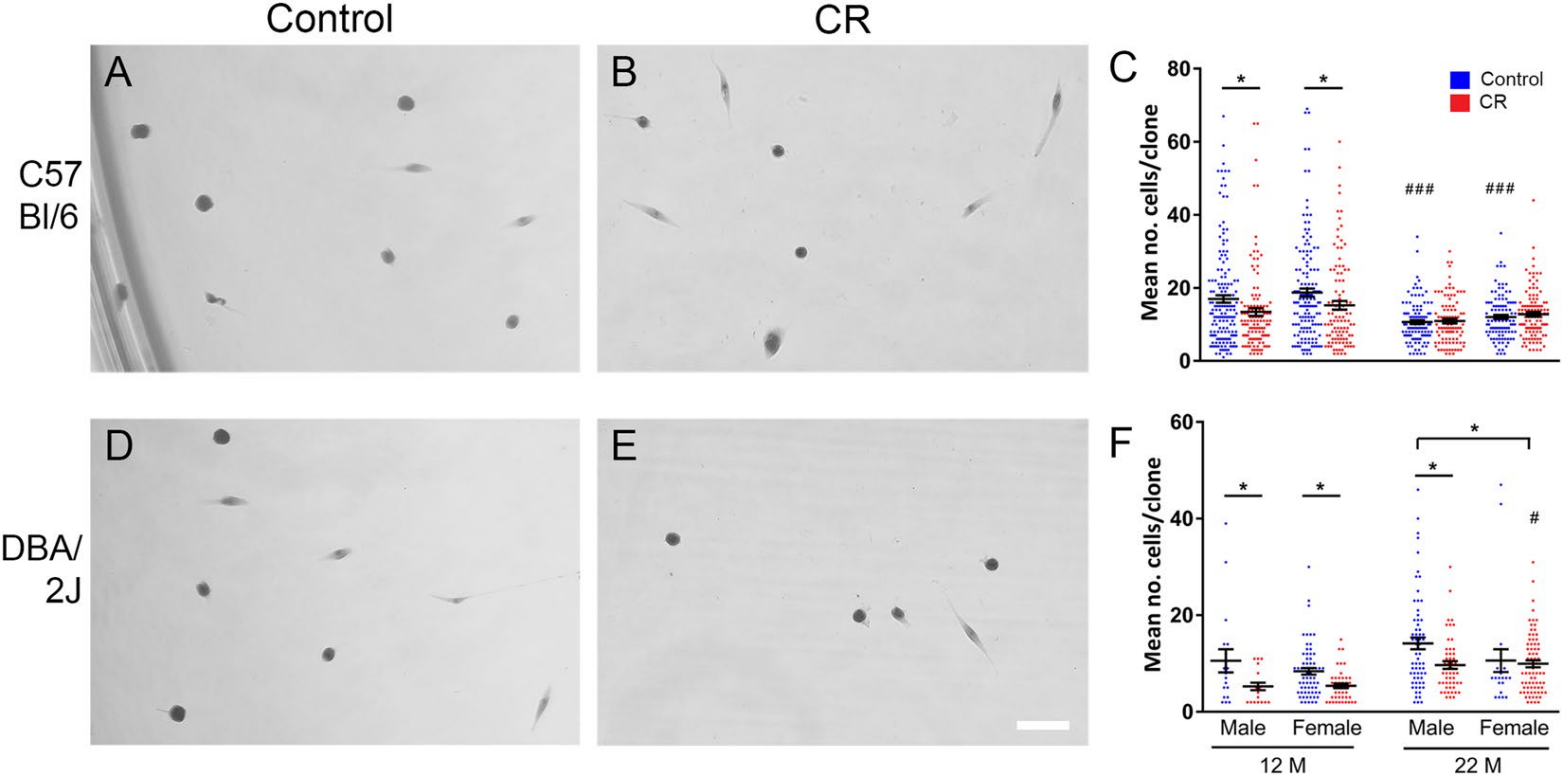
*In vitro* proliferation of satellite cell-derived muscle precursors. Purified satellite cells were prepared by mechanically stripping isolated, intact muscle fibres. Clones were then assessed by plating at 0.5 cells/well in 96 well plates, and culturing for 7 days. Representative images of clones derived from 22 month old C5/B/l6 (**A**,**B**) and DBA/2 (**C**,**D**) mice. Quantification of cell numbers/clone in C57Bl/6 (**C**) and DBA/2 (**F**) mice. n = 23–170 clones per time point/strain/diet; for each, 3–4 mice from each sex were used. Data are presented as mean +/− SEM. ^#^Represents significance between 22 month old mice, and their 12 month counterparts (of same sex, strain and diet).

At 12 months of age, there were significantly more cells/clone derived from satellite cells from EDL muscles of control than CR mice (p < 0.005 for both males and females), but at 22 months of age, there was no significant difference between diets (Fig. 5). However, colonies derived from control mice at 22 months gave rise to fewer cells than their control counterparts at 12 months (p < 0.001 for both male and female cells).

### DBA/2 mice

In clones derived from satellite cells of DBA/2 mice, there were significantly more cells/clone in the control than the CR group at 12 months (p < 0.05 for males and females). At 22 months, a similar result was observed, for males but not females (p < 0.05; Fig. 5).

### Comparison between C57Bl/6 and DBA/2 mice

At 12 months of age, cell numbers were significantly larger in colonies derived from satellite cells of EDL muscles of C57Bl/6 than DBA/2 muscles (p < 0.001). This is in agreement with Fukada *et al*., 2010, who showed that, in contrast to satellite cells from C57Bl/6 mice, satellite cells from DBA/2 mice did not form large colonies^25^. However, the difference in colony size was not significant in 22 month old mice (Fig. 5).

These data indicate that satellite cells derived from CR mice are generally less proliferative *in vitro* than cells derived from control mice. This is the case for cells derived from 12 and 22 month old DBA/2 mice, but only for 12 month old C57Bl/6 mice, suggesting that this difference is strain and time-dependent.

### Calorie restriction affects the ratio of different cellular compartments within skeletal muscle

#### C57Bl/6 mice

There were significantly fewer stromal cells in CR compared to control muscles at 6 months of age (p < 0.01 when sexes are pooled, and <0.05 for females), and at 22 months of age (p < 0.05 for males and when sexes pooled; Table 3).

**Table 3:** Stromal Cells (% of total live, mononucleated cells).

	**Control**			**CR**		
	**Mean**	**± SEM**	**n**	**Mean**	**± SEM**	**n**
**Stromal Cells (% of total live, mononucleated cells)**						
**6 months**						
*Pooled sexes*						
C57BL/6	5.1*	0.4*	7*	3.0*	0.2*	7*
DBA/2	4.9	1.3	6	4.3	1.6	5
*Males*						
C57BL/6	4.5	0.6	4	2.9	0.4	4
DBA/2	5.0	0.1	2	4.2	0.7	2
*Females*						
C57BL/6	5.8	0.2	3	3.0	0.3	3
DBA/2	4.9	0.8	4	4.4	1.3	3
**12 month**						
*Pooled sexes*						
C57BL/6	—	—	—	—	—	—
DBA/2	4.3	0.5	9	3.6	0.2	8
*Males*						
C57BL/6	—	—	—	—	—	—
DBA/2	4.4	0.8	5	3.6	0.3	4
*Females*						
C57BL/6	2.8^&^	1.2^&^	3^&^	2.6	0.9	3
DBA/2	4.3^&^	0.4^&^	4^&^	3.6	0.3	4
**22 month**						
*Pooled sexes*						
C57BL/6	6.3^~ #^	0.4^~ #^	10^~ #^	4.4^~^	0.6^~^	7^~^
DBA/2	8.0^# o^	0.6^# o^	6^# o^	5.5^o^	0.8^o^	7^o^
*Males*						
C57BL/6	6.3^^^	0.6^	7^^^	4.4^^^	1.0^	4^
DBA/2	7.9	0.8	4	6.4	0.9	3
*Females*						
C57BL/6	6.4	0.8	3	4.3	1.0	3
DBA/2	8.3	1.9	2	4.8	1.2	4
**Macrophages (% of total live, mononucleated cells)**						
**6 month**						
*Pooled sexes*						
C57BL/6	0.42*	0.17*	7*	0.28	0.08	7
DBA/2	1.5 *	0.6 *	4 *	0.58	0.3	5
*Males*						
C57BL/6	0.61	0.3	4	0.35	0.1	4
DBA/2	—	—	—	0.20	0.02	3
*Females*						
C57BL/6	0.17^&^	0.06^&^	3^&^	0.18	0.13	3
DBA/2	2.0^&^	0.46^&^	3^&^	1.0	0.9	2
**12 month**						
*Pooled sexes*						
C57BL/6	—	—	—	—	—	—
DBA/2	0.41	0.2	9	0.20	0.04	7
*Males*						
C57BL/6	—	—	—	—	—	—
DBA/2	0.21	0.04	5	0.17	0.02	3
*Females*						
C57BL/6	8.3^~ #^	2_._1^~ #^	3^~ #^	4.6^~^ ^	0.4^~^ ^	3^~^ ^
DBA/2	0.66^#^	0.4^#^	4^#^	0.22^^^	0.7^	4^
**22 month**						
*Pooled sexes*						
C57BL/6	2.1	0.3	9	1.3	0.4	6
DBA/2	2.8^+^	0.5^+^	6^+^	1.1^+^	0.2^+^	7^+^
*Males*						
C57BL/6	2.2^∞^	0.5^∞^	6^∞^	0.9^∞^	0.3^∞^	3^∞^
DBA/2	2.5^w^	0.4^w^	4^w^	1.1^w^	0.2^w^	3^w^
*Females*						
C57BL/6	1.9	0.2	3	1.6	0.6	3
DBA/2	3.3	1.9	2	1.2	0.3	4
**HSCs (% of total live, mononucleated cells)**						
**6 month**						
*Pooled sexes*						
C57BL/6	1.6*	0.2*	6*	0.52 *	0.06 *	6 *
DBA/2	1.1	0.2	5	1.3	0.4	4
*Males*						
C57BL/6	1.4^&^	0.1^&^	4^&^	0.55^&^	0.1^&^	4^&^
DBA/2	1.3	0.1	2	0.8	0.1	2
*Females*						
C57BL/6	1.9	0.7	2	0.45	0.1	2
DBA/2	1.0	0.3	3	2.2	1.0	2
**12 month**						
*Pooled sexes*						
C57BL/6	—	—	—	—	—	—
DBA/2	2.3^~^	0.6^~^	8^~^	0.59^~^	0.1^~^	7^~^
*Males*						
C57BL/6	—	—	—	—	—	—
DBA/2	1.0^#^°	0.1^#^°	4^#^°	0.58°	0.01°	3°
*Females*						
C57BL/6	4.4^^^	2.0^^^	3^^^	0.93^^^	0.8^^^	3^^^
DBA/2	2.8^#+^	0.7^#+^	4^#+^	0.60^+^	0.2^+^	4^+^
**22 month**						
*Pooled sexes*						
C57BL/6	2.8^∞¥^	0.4^∞¥^	9^∞¥^	1.0^∞^	0.3^∞^	6^∞^
DBA/2	5.3^¥°^	0.8^¥°^	6^¥°^	2.3^°^	0.5°	7°
*Males*						
C57BL/6	2.5^¶^	0.3^¶^	6^¶^	1.1	0.5	3
DBA/2	5.4^¶ ≠^	1.1^¶≠^	4^¶ ≠^	2.7^≠^	1.3^≠^	3^≠^
*Females*						
C57BL/6	3.4”	1.0”	3”	1.0”	0.2”	3”
DBA/2	5.3	2.2	2	1.9	0.3	4
**FAPs (% of total live, mononucleated cells)**						
**6 month**						
*Pooled sexes*						
C57BL/6	4.4*	0.3*	7*	2.4*	0.2*	7*
DBA/2	3.5	0.6	6	2.7	0.5	5
*Males*						
C57BL/6	4.1	0.5	4	2.4	0.4	4
DBA/2	4.7	0.4	2	3.5	0.3	2
*Females*						
C57BL/6	5.1^& ~^	0.1^& ~^	3^& ~^	2.4^&^	0.3^&^	3^&^
DBA/2	2.9^~^	0.8^~^	4^~^	2.1	0.8	3
**12 month**						
*Pooled sexes*						
C57BL/6	—	—	—	—	—	—
DBA/2	3.9	0.4	9	3.1	0.2	8
*Males*						
C57BL/6	—	—	—	—	—	—
DBA/2	3.9	0.6	5	2.9	0.2	4
*Females*						
C57BL/6	2.4^+^	0.9^+^	3^+^	1.9^∞^	0.6^∞^	3^∞^
DBA/2	3.9^+^	0.3^+^	4^+^	3.2^∞^	0.3^∞^	4^∞^
**22 month**						
*Pooled sexes*						
C57BL/6	5.1^¥^	0.5^¥^	10^¥^	2.5^¥^°	0.2^¥^°	7^¥^°
DBA/2	6.0^¶^	0.8^¶^	6^¶^	4.2°^¶^	0.6°^¶^	7°^¶^
*Males*						
C57BL/6	5.1^≠^	0.7^≠^	7^≠^	2.5^≠^°	0.2^≠^°	4^≠^°
DBA/2	5.4	0.9	4	4.7°	0.6°	3°
*Females*						
C57BL/6	5.0^=^	0.9^=^	3^=^	2.6^=^	0.3^=^	3^=^
DBA/2	7.4	2.3	2	3.9	1.0	4

Populations of muscle-resident cells quantified by FACS. Cells were isolated from disaggregated muscle and analysed by FACS in accordance with the scheme presented in Supplementary Figure 1. Frequency is expressed as percentage of cells out of the total live/singlet cell count. Data are presented as mean ± standard error of the mean (SEM), and n numbers are specified in each case. Superscript symbols and underlinings are used to match measurements that are significant between each other.

CR also reduced the proportions of HSCs, macrophages and FAPs within skeletal muscle. HSCs were reduced at 6 months (p < 0.01 when sexes pooled and <0.05 for males), at 12 months (p < 0.05 for females) and at 22 months (p < 0.01 when sexes pooled and <0.05 for females). Macrophages were reduced at 12 months (p < 0.05 for females) and at 22 months (p < 0.05 for males). FAPs were reduced at 6 months (p < 0.01 when sexes pooled, and p < 0.05 for both males and females), and at 22 months (p < 0.01 when sexes pooled and <0.05 for both males and females).

### DBA/2 mice

There were significantly fewer stromal cells in CR compared to control muscles at 22 months (p < 0.05 when sexes pooled). CR also reduced the proportions of HSCs, macrophages and FAPs. HSCs were reduced at 12 months (p < 0.01 when sexes pooled and <0.05 for both males and females) and at 22 months (p < 0.05 when sexes pooled and for males). Macrophages were reduced at 6 months (p < 0.05 when sexes pooled) and at 22 months (p < 0.01 when sexes pooled and <0.05 for males). FAPs were reduced at 22 months (p < 0.05 when pooled).

### Comparison between C57Bl/6 and DBA/2 mice

Differences were also observed as animals aged. For C57Bl/6 mice there were significant increases in macrophages and HSCs by 22 months relative to 6 months; in DBA/2 mice, increases in stromal cells, macrophages, HSCs and FAPs were apparent at 22 months relative to 6 and 12 months.

All in all, although strain and age-dependent for some cell types, these data suggest that CR has the capacity to reduce stromal cell, macrophage, HSC and FAP numbers, including that which occurs during normal ageing.

Multiple regression analysis confirms the findings that CR is able to reduce stromal cell, macrophage, HSC, and FAPS numbers in skeletal muscle (Supplementary Table 3). Stromal cell numbers were primarily determined by age (ß = 0.628; p < 0.0005), but were reduced by increasing time on CR (ß = −0.418; p < 0.0005). Strain had the smallest impact with DBA mice having a higher number of stromal cells than C57Bl/6 mice (ß = −0.204; p < 0.05). The proportion of macrophages was also determined mainly by age (ß = 0.422; p < 0.01); time on calorie restriction (ß = −0.319; p < 0.05); strain (ß = 0.264; p < 0.05) with C57Bl/6 mice having elevated numbers compared to DBAs; and sex (ß = 0.26; p < 0.05) where females displayed a higher number of macrohpages than males. For the remainder of the cell types analysed, age and time on calorie restriction were the only significant factors influencing the proportion of these cell types in skeletal muscle. The proportion of HSCs was primarily affected by age (ß = 0.702; p < 0.0005) as were FAPS (ß = 0.576; p < 0.0005). This was followed by time on CR for HSCs (ß = −0.584; p < 0.0005) and FAPS (ß = −0.524; p < 0.0005).

Together these analysis show that the proportion of these cell types increase with age, but are reduced by increasing time spent on CR. Strain makes a minor difference to the amount of stromal cells, whilst the proportion of macrophages is also affected by strain and sex.

### A reduction in satellite cell number does not increase the extent of fibrosis

**Figure 6:**
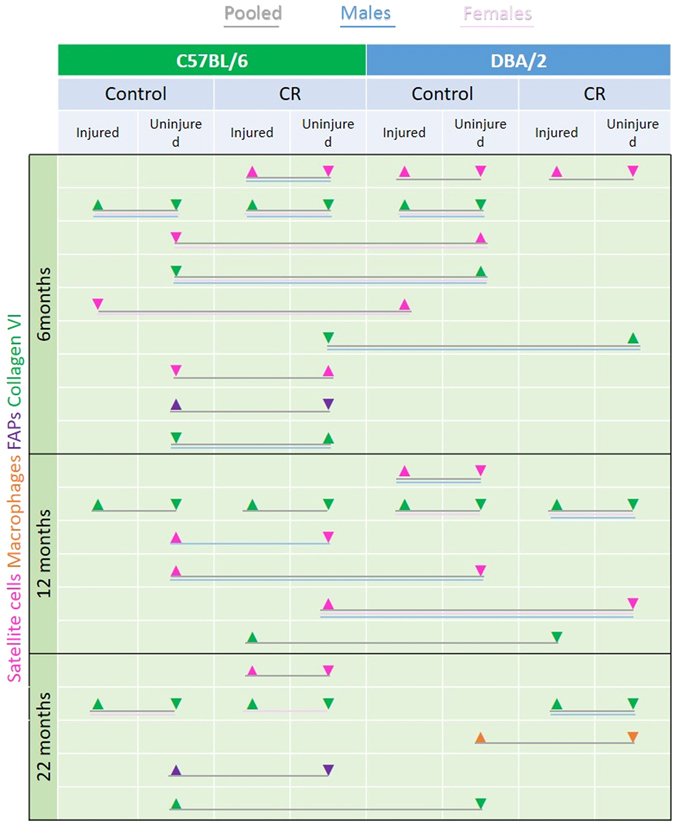
Summary of changes in satellite cells (pink), macrophages (brown), FAPS (purple) and collagen VI (green) in male, female and pooled CR and control C57Bl/6 and DBA/2 mice at different timepoints. Arrows signify a significant increase or decrease at 6, 12 and 22 months of age (after 2.5, 8.5 and 18.5 months of CR).

There is evidence that a loss of satellite cells causes an increase in muscle fibrosis^44–46^, so we correlated the increase or decrease in satellite cell number with increase or decrease collagen VI within the muscles (Fig. 6). At the 6 month timepoint, both satellite cell number and collagen VI were significantly increased in injured compared with non-injured muscles of CR C57Bl/6 mice and in in injured compared with non-injured muscles of control DBA/2 mice. Both satellite cell number and collagen VI were significantly greater in uninjured muscles of CR than control C57Bl/6 mice, but FAPS were significantly reduced in CR than control muscles of these mice. Satellite cell number and collagen VI were significantly increased in non-injured control DBA/2 compared with non-injured control C57B/6 muscles.

At the 12 month timepoint, CR reduced the number of satellite cells in non-injured male C57Bl/6 muscles, but this was not associated with a significant change in the quantity of collagen VI. All in all, our model shows no clear indication that a reduction in satellite cell number is associated with an increase in fibrosis; it is likely that this process is modulated by a variety of cell types and/or signaling factors.

## Discussion

Our overall aim was to investigate the effect of calorie restriction on skeletal muscle stem cells in ageing mice of two different strains - C57Bl/6, in which calorie restriction increases longevity, and DBA/2, in which it does not^24^. We found that CR modulates the satellite cell number (increased at 6, but decreased at 12 months), and increases collagen VI content of mouse muscle. CR also reduces the size of clones *in vitro* from satellite cells derived from these muscles, and delays the regenerative response. CR affects the percentage of stromal cells, macrophages, HSCs and FAPs in skeletal muscle. But these changes are not consistent over time and between strain and sex.

C57Bl/6 is a commonly used mouse for many studies, including ageing, response to dietary intervention^47^ and studies on satellite cells and muscle regeneration^8, 20, 48, 49^, but it should be borne in mind that there may be mutations that act as genetic modifiers to affect the outcome of experiments^50^. Studies on muscle regeneration have been done on C57Bl/6 mice of different sexes^51–54^. Studies on ageing have been mostly done on male C57Bl/6 mice^47, 55, 56^.

Cerletti *et al*.^20^ used male C57Bl/6 mice and initiated short-term CR at either 2 or 18 months of age. After 12 weeks, satellite cells were prepared from extensor digitorum longus, gastrocnemius, quadriceps, soleus, tibialis anterior, and triceps brachii muscles and they found a significantly greater frequency of satellite cells (by FACS analysis; satellite cells/g of muscle) and a greater number of Pax7+ satellite cells on isolated fibres prepared from TA muscles from CR compared to control mice. Their data suggested that modulation of mitochondrial bioenergetics is a mechanism by which CR enhances satellite cell frequency and activity in skeletal muscle. Our study differs from that of Cerletti *et al*., as they investigated short term (3 months) CR starting at either 2 or 18 months of age, whereas we examined short and long-term CR throughout the normal lifespan – (6, 12 and 22 months of age, corresponding to 2.5, 8.5 and 18.5 months of CR). Cerletti *et al*. used only male C57BL/6 mice, whereas we used males and females from two strains (C57Bl/6 and DBA/2). Additional parameters we examined included skeletal muscle fibrosis and various muscle-resident cell types, including endothelial cells, stromal cells, FAPs and HSCs (Cerletti *et al*. also examined the latter cell type, and our results confirmed their findings that CR reduces their numbers in muscle).

Our findings in C57Bl/6 (data pooled from male and female mice) after 3 months of calorie restriction (6 month old mice) are in agreement with Cerletti *et al*., in that we found more satellite cells in TA muscles of CR than control mice. But when we looked at later timepoints and also in a different mouse strain (DBA/2), there were either no significant differences, or in some cases a reduction, in the number of satellite cells in response to CR. We also found that satellite cells derived from muscles of CR mice were less proliferative than those from control (at 12 months in C57Bl/6 and at 12 and 22 months in DBA/2 mice). The differences in clonogenicity between control and CR satellite cells suggest that there are cell-intrinsic effects that persist even after they have been removed from their *in vivo* environments and cultured for 7 days; such changes could be encoded via epigenetic mechanisms, or long-lasting changes in levels of transcription factors and/or signaling/cell cycle proteins. All in all, our strain, time and sex-dependent findings suggest that the beneficial effects of CR on satellite cell number and function may be less clear-cut than originally supposed. The length of time of CR and the genetic background may both significantly modify the satellite cell response to CR.

CR resulted in a reduction in fibre size 7 days after injury, compared to dietary control mice (observed in 6 month female C57Bl/6 mice and in 22 month DBA/2 mice strain and sex-matched control mice). This suggests that regeneration is delayed as a response to CR^49^, but why this did not occur at other timepoints is not clear. The notexin injection in these 22 month CR mice elicited less widespread TA muscle injury than in the 6 month CR mice, which may explain the difference. It might be that myofibres are reduced in size as a response to CR and the consequent reduction in nutrients, i.e. there are the same number of fibres in CR mice, but they take longer to increase in size, particularly during regeneration.

Fibrosis was increased in non-injured muscles of 6 month CR C57Bl/6 compared to control mice, but not at the later timepoints and not in DBA/2 mice. These findings are complicated by the fact that there is more fibrosis in non-injured muscles of DBA/2 than C56Bl/6 mice at 6 months of age. The marker of fibrosis that we used was collagen VI^29^ and there is evidence that it plays a part in muscle regeneration^42^, suggesting that its increase in non-injured muscles of CR mice might be beneficial rather than detrimental. It should be noted that collagen VI is a normal component of the basal lamina as well as being a marker of fibrosis.

It has been suggested that a loss of satellite cells causes an increase in muscle fibrosis during muscle hypertrophy either in response to overload^44–46^, or to stretch^57^, or with increasing age^58^. But in these experiments, the reduction in satellite cell number was from a relatively young age (2 or 4 months of age) and the mice were on a mixed genetic background (C57Bl/6-129). Our results show no clear alignment with these previous findings, suggesting that fibrosis is modulated by various cell types and/or chemical factors, beyond those attributed to satellite cells. It is interesting that CR resulted in reduced numbers of FAPS but increased fibrosis in uninjured C57Bl/6 mice at 6 months, since this cell type might be expected to produce fibrotic material; again this suggests multiple influencing factors in this process^59^.

We also found a significant decrease in the percentage of stromal cells, macrophages, HSCs (CD45+ cells, that might include side population (SP) cells) and FAPs in the cell populations extracted from muscles of CR than control mice. These cells have accessory roles in muscle regeneration, affecting satellite cell proliferation and differentiation (reviewed^60^). Macrophages invade injured muscle: they are at first pro-inflammatory, but then switch to being anti-inflammatory as the muscle degeneration-regeneration process progresses^61^ (reviewed^62^). There is evidence that SP cells can contribute to muscle regeneration^63^, but their number and function in ageing muscle have not been studied. But there are reduced numbers of CD45+ cells in regenerating areas of skeletal muscle with increasing age^64^.

Stromal cells contribute to the ECM and also secrete growth factors that influence muscle regeneration (reviewed^65, 66^). FAPs, that have both fibroblastic and adipogenic potential, are a source of trophic factors and seem to play a role in muscle regeneration^38, 67^. However, the role of these different cell types in the homeostasis of non-injured adult muscles is not known^68^. In addition, others have suggested that FAPs might contribute to the fat infiltration that occurs with ageing, as they do in pathological states such as muscular dystrophy and muscle disuse (reviewed in ref. 69). The increased collagen VI, increased satellite cell number, decrease in the percentage of stromal cells, macrophages, HSCs and FAPs after 3 months of CR may act in concert to improve satellite cell function and muscle regeneration. In addition, the reduction in FAP numbers may have a protective effect on the muscle itself, given the link between these cells and fat infiltration. But why these changes do not all persist with time and the dissimilarities between the 2 mouse stains, combined with sex differences, urge caution in applying CR to improve skeletal muscle function across the lifespan in humans.

We found clear differences in skeletal muscle between the two mouse strains. Non-injured TA muscles of 6 month old DBA/2 mice have significantly more satellite cells than C57Bl/6. In contrast, but similar to our data on 12 and 22 month old mice, Fukada *et al*. found no difference in satellite cell number in TA muscles of 6 week old DBA/2 and C57Bl/6 mice. We found that 6 month old male DBA/2 mice have smaller diameter muscle fibres than do C57Bl/6 mice. The non-injured TA muscles of our DBA/2 mice were more fibrotic than C57Bl/6 mice at 6 months of age, in agreement with Fukada *et al*.^25^.

Differences in quantitative trait loci (QTL) that affect HSC number have been found in C57Bl/6 and DBA/2 mice^70, 71^. There was differential expression of Slit2 between the two mouse strains, which may explain the lower number of bone marrow-derived HSCs in C57Bl/6 mice^71^, but we did not see any change in the percentage of HSCs in the single-cell populations derived from skeletal muscle. Interestingly, Slit2 modifies angiogenesis in skeletal muscle^72^; although there are no reports that Slit2 is expressed in satellite cells, its overexpression does lead to an increase in numbers of other types of stem cells (HSCs, intestinal stem cells). It is tempting to speculate that genetic differences such as this might be responsible for the increased number of satellite cells in DBA/2 compared to C57Bl/6 mice at 6 months of age.

The relationship between environmental variables such as CR and genetics, sex, and ageing is complex. For example, the effects of CR on reducing tumour formation in C57Bl/6 and DBA/2 mice are different^73^ and, like our study, they found that the effects of CR were inconsistent and variable, depending on strain background and sex. Similarly, Li *et al*. found differences in mitochondrial oxidative damage between C57Bl/6 and DBA/2 mice^74^, but the association between ageing, oxidative damage and CR did not follow a uniform pattern in different tissues and genotypes. Recently, Mitchell *et al*. demonstrated a strain-, sex-, and dose-dependent effect of CR on health and survival in C57Bl/6 and DBA/2 mice^18^.

The decline in sex hormones with increasing age is a major variable that may be affecting our findings. Estrogens have been shown to increase the response of satellite cells to injury or eccentric exercise and testosterone promotes muscle regeneration after injury. Both androgens and estrogens promote satellite cell activation and proliferation and stimulate expression of growth-promoting genes^75^ (reviewed^76^) and also play a role in satellite cell self-renewal following muscle injury^77^. Whether CR has any beneficial effect when started on young mice with reduced levels of sex hormones (e.g. ovariectomized females and castrated males), or whether testosterone or oestrogen implants would maintain a clear benefit of CR into old age would be of interest when considering the use of CR for prevention of sarcopenia in humans.

Using cells prepared from the same mice used for the studies on skeletal muscle that we describe here, we found that CR preserves a naïve T cell phenotype and an immature NK cell phenotype as mice age. But the effects of calorie restriction on lymphocyte maturation were more marked in C57Bl/6 than in DBA/2 mice indicating that delayed lymphocyte maturation correlates with extended lifespan^78^. In addition, we examined the bone marrows of the same mice and found that CR also significantly delays the age-based accumulation of mitotically-inactive HSCs (Beaver *et al*., in preparation). That CR has such different effects on hematopoietic and skeletal muscle stem cell systems within the same mouse is intriguing and of profound interest for any future applications of CR in humans.

## Electronic supplementary material

Supplementary information
