# Supplementary material for: The effect of calorie restriction on mouse skeletal muscle is sex, strain and time-dependent
Source: Sci Rep. 2017 Jul 11;7:5160. doi: 10.1038/s41598-017-04896-y (PMC5505993; doi:10.1038/s41598-017-04896-y)
Supplement: Supplementary file 1 — Supplementary information [file 41598_2017_4896_MOESM1_ESM.doc]

**The effect of calorie restriction on mouse skeletal muscle is sex, strain and time-dependent.**

**Luisa Boldrin^1+^, Jacob A Ross^1+^, Charlotte Whitmore^1^, Bruno Doreste^1^, Charlotte Beaver^2^, Ayad Eddaoudi^3^, Daniel J Pearce^2^, Jennifer E Morgan^1*^**

^1^ Dubowitz Neuromuscular Centre, Molecular Neurosciences Section, Developmental Neurosciences Programme, UCL Great Ormond Street Institute of Child Health, 30 Guilford Street, London, WC1N1EH, UK

^2^UCL Institute of Healthy Ageing, University College London, London WC1E 6BT, UK

^3^Flow Cytometry Core Facility, UCL Great Ormond Street Institute of Child Health, Camelia Botnar Laboratories, Great Ormond Street Hospital, 85 Lamb's Conduit, London, WC1N 3JH, UK

**Figure legends**.

**Supplementary Figure 1**.

Analysis scheme of FACS-sorted cell populations.

FACS plots from a control female C57Bl/6 mouse at 22 months, to demonstrate how the FACS plots were analysed. Live singlet cells were gated based on DAPI fluorescence and size. Following this, macrophages and haematopoietic stem cells (HSCs) were detected with CD68 and CD45 antibodies, respectively (bottom right panel). For analysis of other cell populations, lineage (Lin) negative fractions were taken, and gated for CD31+/Sca1+ (endothelial cells), CD31-/Sca1+ (stromal cells), and CD31-/Sca1+/CD34+/integrin-α7- (fibro/adipogenic precursors, FAPs). Individual populations were then expressed as % of total live/singlet cell count.

**Supplementary Table 1**.

Comparison of distributions of the myofibre cross sectional area (CSA) in injured and un-injured control and CR muscles.

Distributions of the myofibre cross sectional area (CSA) in control and CR muscles of injured right TA (RTA) or uninjured left TA (LTA) were compared by Chi-Square. Significant values are highlighted in bold.

**Supplementary Table 2.**

Simple multiple regression summary table of factors affecting median fibre CSA.

**Supplementary Table 3.**

Summary of simple multiple regression analysis for variables influencing the proportion of individual cell types in skeletal muscle.

**Supplementary Table 1**


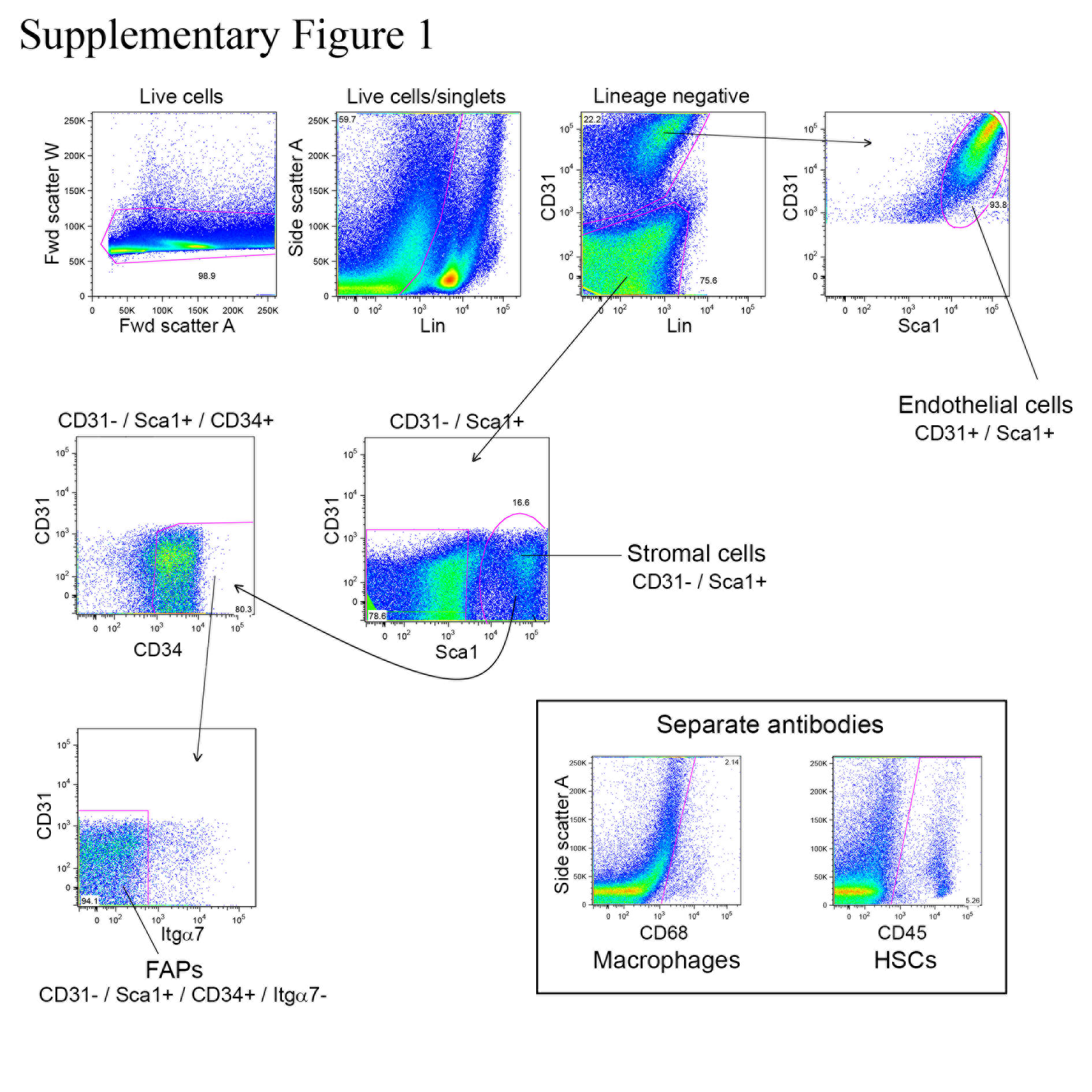


**Supplementary Table 1**

**CSA**

| **6 months** | | | | | | | | |
| --- | --- | --- | --- | --- | --- | --- | --- | --- |
|  | **Control vs CR injured (RTA)** | | | | **Control vs CR uninjured (LTA)** | | | |
|  | **Chi-square** | **p value** | **n control** | **n CR** | **Chi-square** | **p value** | **n control** | **n CR** |
| **C57BL/6** | 7.22 | 0.61 | 12 | 12 | 2.82 | 0.97 | 12 | 12 |
| **DBA/2** | 2.93 | 0.98 | 12 | 12 | 10.32 | 0.33 | 12 | 12 |
|  |  |  |  |  |  |  |  |  |
| **Males** | **Control vs CR injured (RTA)** | | | | **Control vs CR uninjured (LTA)** | | | |
|  | **Chi-square** | **p value** | **n control** | **n CR** | **Chi-square** | **p value** | **n control** | **n CR** |
| **C57BL/6** | 3.54 | 0.94 | 6 | 6 | 5.09 | 0.83 | 6 | 6 |
| **DBA/2** | 1.92 | 1.00 | 6 | 6 | 8.63 | 1.00 | 6 | 6 |
|  |  |  |  |  |  |  |  |  |
| **Females** | **Control vs CR injured (RTA)** | | | | **Control vs CR uninjured (LTA)** | | | |
|  | **Chi-square** | **p value** | **n control** | **n CR** | **Chi-square** | **p value** | **n control** | **n CR** |
| **C57BL/6** | 21.02 | **0.01** | 6 | 6 | 1.92 | 0.99 | 6 | 6 |
| **DBA/2** | 5.18 | 0.82 | 6 | 6 | 11.89 | 0.22 | 6 | 6 |
|  |  |  |  |  |  |  |  |  |
|  |  |  |  |  |  |  |  |  |
| 12 months | | | | | | | | |
|  | **Control vs CR injured (RTA)** | | | | **Control vs CR uninjured (LTA)** | | | |
|  | **Chi-square** | **p value** | **n control** | **n CR** | **Chi-square** | **p value** | **n control** | **n CR** |
| **C57BL/6** | 11.60 | 0.24 | 8 | 10 | 6.94 | 0.64 | 11 | 12 |
| **DBA/2** | 7.14 | 0.62 | 10 | 6 | 10.57 | 3.54 | 13 | 10 |
|  |  |  |  |  |  |  |  |  |
| **Males** | **Control vs CR injured (RTA)** | | | | **Control vs CR uninjured (LTA)** | | | |
|  | **Chi-square** | **p value** | **n control** | **n CR** | **Chi-square** | **p value** | **n control** | **n CR** |
| **C57BL/6** | 12.96 | 0.16 | 5 | 5 | 7.89 | 0.54 | 6 | 6 |
| **DBA/2** | 18.69 | **0.03** | 4 | 3 | 16.13 | **0.06** | 6 | 5 |
|  |  |  |  |  |  |  |  |  |
| **Females** | **Control vs CR injured (RTA)** | | | | **Control vs CR uninjured (LTA)** | | | |
|  | **Chi-square** | **p value** | **n control** | **n CR** | **Chi-square** | **p value** | **n control** | **n CR** |
| **C57BL/6** | 7.87 | 0.55 | 3 | 5 | 6.72 | 0.67 | 5 | 6 |
| **DBA/2** | 2.77 | 0.97 | 6 | 3 | 9.48 | **0.39** | 7 | 5 |
|  |  |  |  |  |  |  |  |  |

|  |  |  |  |  |  |  |  |  |
| --- | --- | --- | --- | --- | --- | --- | --- | --- |
| **22 months** | | | | | | | | |
|  | **Control vs CR injured (RTA)** | | | | **Control vs CR uninjured (LTA)** | | | |
|  | **Chi-square** | **p value** | **n control** | **n CR** | **Chi-square** | **p value** | **n control** | **n CR** |
| **C57BL/6** | 8.34 | 0.50 | 9.0 | 7.0 | 5.34 | 0.80 | 10 | 10 |
| **DBA/2** | 4.75 | 0.86 | 5.0 | 6.0 | 6.05 | 0.73 | 6 | 6 |
|  |  |  |  |  |  |  |  |  |
| **Males** | **Control vs CR injured (RTA)** | | | | **Control vs CR uninjured (LTA)** | | | |
|  | **Chi-square** | **p value** | **n control** | **n CR** | **Chi-square** | **p value** | **n control** | **n CR** |
| **C57BL/6** | 7.83 | 0.55 | 5 | 3 | 10.67 | 0.30 | 6 | 4 |
| **DBA/2** | 2.01 | 0.99 | 3 | 4 | 11.83 | 0.22 | 4 | 3 |
|  |  |  |  |  |  |  |  |  |
| **Females** | **Control vs CR injured (RTA)** | | | | **Control vs CR uninjured (LTA)** | | | |
|  | **Chi-square** | **p value** | **n control** | **n CR** | **Chi-square** | **p value** | **n control** | **n CR** |
| **C57BL/6** | 7.15 | 0.62 | 4 | 4 | 1.91 | 0.99 | 4 | 6 |
| **DBA/2** | 14.78 | **0.10** | 2 | 2 | 4.69 | 0.86 | 2 | 3 |
|  |  |  |  |  |  |  |  |  |
|  |  |  |  |  |  |  |  |  |
|  |  |  |  |  |  |  |  |  |
|  | |  |  |  |  |  |  |  |

**Supplementary Table 2**

| Simple multiple regression summary table of factors affecting median fibre CSA | | | | |
| --- | --- | --- | --- | --- |
| Variable | *B* | *SE B* | ß | Sig. |
| Injury (Y/N) | 1.75 E-4 | 1.4E-5 | 0.624 | 0.000 |
| Strain | -3.73E-05 | 1.4E-5 | -0.132 | 0.009 |
| Sex | -3.04E-05 | 1.4E-5 | -0.109 | 0.031 |
| Time on CR | -1.41E-06 | 3.26E-7 | -0.248 | 0.000 |
| Age | 5.03E-06 | 1E-6 | 0.226 | 0.000 |
|  |  |  |  |  |
| R^2^ | 0.41 | | | |
| F | 38.704** | | | |
| N | 226 | | | |
| ** p<0.0005 |  |  |  |  |

**Supplementary Table 3**

| Summary of simple multiple regression analysis for variables influencing the proportion of individual cell types in skeletal muscle | | | | | | | | | | | | | | | | | | | | | | | | | | | | |
| --- | --- | --- | --- | --- | --- | --- | --- | --- | --- | --- | --- | --- | --- | --- | --- | --- | --- | --- | --- | --- | --- | --- | --- | --- | --- | --- | --- | --- |
| Variable | Stromal Cells | | | | | Macrophages | | | | | | | HSCs | | | | | | | FAPS | | | | | | | | |
|  | *B* | *SE B* | ß | Sig. | | *B* | *SE B* | | ß | | Sig. | | *B* | *SE B* | | ß | | Sig. | | *B* | *SE B* | | ß | | Sig. | |  | |
| Strain | -0.82 | 0.384 | -0.204 | 0.037 | | 1.062 | 0.433 | | 0.264 | | 0.017 | | -0.359 | 0.344 | | -0.099 | | 0.301 | | -0.576 | 0.335 | | -0.169 | | 0.090 | |  | |
| Sex | -0.435 | 0.386 | -0.108 | 0.264 | | 1.046 | 0.434 | | 0.26 | | 0.019 | | 0.378 | 0.345 | | 0.105 | | 0.278 | | -0.415 | 0.336 | | -0.122 | | 0.221 | |  | |
| Time on CR | -0.03 | 0.008 | -0.418 | 0.000 | | -0.023 | 0.009 | | -0.319 | | 0.013 | | -0.037 | 0.007 | | -0.584 | | 0.000 | | -0.031 | 0.007 | | -0.524 | | 0.000 | |  | |
| Age | 0.185 | 0.033 | 0.628 | 0.000 | | 0.125 | 0.037 | | 0.422 | | 0.001 | | 0.187 | 0.029 | | 0.702 | | 0.000 | | 0.145 | 0.029 | | 0.576 | | 0.000 | |  | |
|  |  |  |  |  | |  |  | |  | |  | |  |  | |  | |  | |  |  | |  | |  | |  | |
| R^2^ | 0.369 | | | | | 0.262 | | | | | | | 0.448 | | | | | | | 0.355 | | | | | | | | |
| F | 10.099** | | | | | 5.688* | | | | | | | 12.380** | | | | | | | 9.210** | | | | | | | | |
| N | 74 | | | | | 69 | | | | | | | 66 | | | | | | | 74 | | | | | | | | |
| * p<0.001 | **p<0.0005 | |  | |  |  | |  | |  | |  |  | |  | |  | |  |  | |  | |  | |  | |  |
